# Whole genome analysis reveals the diversity and evolutionary relationships between necrotic enteritis-causing strains of *Clostridium perfringens*

**DOI:** 10.1186/s12864-018-4771-1

**Published:** 2018-05-22

**Authors:** Jake A. Lacey, Theodore R. Allnutt, Ben Vezina, Thi Thu Hao Van, Thomas Stent, Xiaoyan Han, Julian I. Rood, Ben Wade, Anthony L. Keyburn, Torsten Seemann, Honglei Chen, Volker Haring, Priscilla A. Johanesen, Dena Lyras, Robert J. Moore

**Affiliations:** 1 Infection and Immunity Program, Monash Biomedicine Discovery Institute and Department of Microbiology, Monash University, Clayton, VIC 3800 Australia; 2 CSIRO Biosecurity Flagship, Australian Animal Health Laboratory, Geelong, VIC 3220 Australia; 3 Poultry Cooperative Research Centre, University of New England, Armidale, NSW 2351 Australia; 4 School of Medicine, Deakin University, Waurn Ponds, VIC 3216 Australia; 5 Victorian Life Sciences Computation Initiative, University of Melbourne, Parkville, VIC 3010 Australia; 6 School of Science, RMIT University, Bundoora, VIC 3083 Australia

**Keywords:** *Clostridium perfringens*, Necrotic enteritis, Capsule, Adhesion, Prophage, Genome, Pangenome

## Abstract

**Background:**

*Clostridium perfringens* causes a range of diseases in animals and humans including necrotic enteritis in chickens and food poisoning and gas gangrene in humans. Necrotic enteritis is of concern in commercial chicken production due to the cost of the implementation of infection control measures and to productivity losses. This study has focused on the genomic analysis of a range of chicken-derived *C. perfringens* isolates, from around the world and from different years. The genomes were sequenced and compared with 20 genomes available from public databases, which were from a diverse collection of isolates from chickens, other animals, and humans. We used a distance based phylogeny that was constructed based on gene content rather than sequence identity. Similarity between strains was defined as the number of genes that they have in common divided by their total number of genes. In this type of phylogenetic analysis, evolutionary distance can be interpreted in terms of evolutionary events such as acquisition and loss of genes, whereas the underlying properties (the gene content) can be interpreted in terms of function. We also compared these methods to the sequence-based phylogeny of the core genome.

**Results:**

Distinct pathogenic clades of necrotic enteritis-causing *C. perfringens* were identified. They were characterised by variable regions encoded on the chromosome, with predicted roles in capsule production, adhesion, inhibition of related strains, phage integration, and metabolism. Some strains have almost identical genomes, even though they were isolated from different geographic regions at various times, while other highly distant genomes appear to result in similar outcomes with regard to virulence and pathogenesis.

**Conclusions:**

The high level of diversity in chicken isolates suggests there is no reliable factor that defines a chicken strain of *C. perfringens*, however, disease-causing strains can be defined by the presence of *netB*-encoding plasmids. This study reveals that horizontal gene transfer appears to play a significant role in genetic variation of the *C. perfringens* chromosome as well as the plasmid content within strains.

## Background

*Clostridium perfringens* is a bacterial pathogen that causes a wide variety of diseases ranging from gas gangrene and food poisoning in humans to necrotic enteritis in chickens [1]. Aside from host factors, the type and severity of disease is dependent on the production of a set of toxins and extracellular enzymes [1]. Most *C. perfringens* toxins are not produced by all strains; their structural genes are present as a part of the accessory genome encoded on large conjugative plasmids [2, 3]. These plasmids are found in various combinations within different strains [4–7]. Some plasmid-encoded toxins have been uniquely associated with a specific disease; for example, NetB is associated with necrotic enteritis. Other toxins, such as the beta2 and beta toxins, are encoded on highly variable plasmids and are produced by strains associated with several diseases [2, 8, 9]. By contrast, alpha toxin is chromosomally encoded and is carried by all *C. perfringens* strains.

Classification of *C. perfringens* strains has traditionally been based on the profile of four typing toxins. Currently, strains are classified as type A to type E based on presence or absence of the toxins. Necrotic enteritis is caused by type A strains that produce NetB, a pore-forming toxin that is essential for disease in chickens [10]. Type A strains, which carry the alpha toxin gene, but none of the other toxin typing genes, also cause several other diseases, including gas gangrene and bovine abomasitis. Type A strains are also normal members of the gut microbiota in animals and humans, as well as being found ubiquitously in the environment [11–13].

A genomic comparison of three type A strains showed that there is a significant degree of genomic variation between strains SM101, ATCC13124 and 13, with over 300 genomic islands variably present, almost all of which are chromosomally encoded [14]. This chromosomal variation is also evident from studies that have used pulse-field gel electrophoresis (PFGE) to examine genomes via restriction digest fingerprinting. Strains from similar origins and disease-causing backgrounds are highly variable and have unique fingerprints, suggesting a highly variable genomic content in strains even when they appear to cause the same disease [15–17]. A whole-genome comparison of 12 strains from different origins revealed that type A strains could not be differentiated from the types B to E by examining the chromosomal gene content. Only the presence of plasmid-encoded genes allowed a clear differentiation between the different toxinotypes [18].

Necrotic enteritis is a disease of concern to the poultry industry due to the negative impact that it has on production costs. Necrotic enteritis reduces the efficiency of feed conversion and in severe cases causes death [19–21]. The disease is prevalent worldwide and, in addition to its significant economic impact on the poultry industry, it represents an important animal welfare issue. The NetB toxin is an essential virulence factor in necrotic enteritis [10], and the gene encoding this toxin is located on a pathogenicity locus, NEloc-1, which is in turn encoded on a large conjugative plasmid [7, 10, 22–25]. Some necrotic enteritis strains also produce another toxin, TpeL, which is also encoded on large plasmids; however, the role of this toxin, if any, in necrotic enteritis is unknown [26, 27]. Necrotic enteritis strains also sometimes encode resistance to the antibiotics tetracycline and bacitracin on plasmids, once again emphasising the importance of plasmids in the phenotypic differentiation of *C. perfringens* strains [7, 28].

Sub-genomic analyses involving multi-locus sequence typing (MLST) has identified several subtypes or clonal complexes of poultry-associated *C. perfringens* isolates [15, 23]. Two of these subtypes are predominately composed of pathogenic strains, which are defined by the presence of *netB* [23]. A comparative genome hybridization (CGH) microarray study [23] showed that among the 54 chicken isolates examined, approximately 400 genes were variably present. The variable genes were organised into 142 genomic regions, 49 of which were associated with *netB*-positive isolates [23]. Some of the variation was associated with chromosomal regions with predicted roles in metabolism, fitness associated factors, and plasmid maintenance. The CGH study revealed clustering of necrotic enteritis-associated strains into two distinct subtypes, a CP4-like and JGS4143-like subtype [23].

In this study, whole genome sequencing (WGS) was performed on a diverse collection of poultry-derived *C. perfringens* isolates from both necrotic enteritis-affected and healthy birds. The genomes were compared using whole genome content analysis to investigate the level of chromosomal variation. Since the role of plasmids in *C. perfringens* has been well established, and they are known to be transferable between strains via lateral gene transfer, this investigation focused on chromosomal variation.

## Results

### Strain selection and sequence statistics

**Fig. 1:**
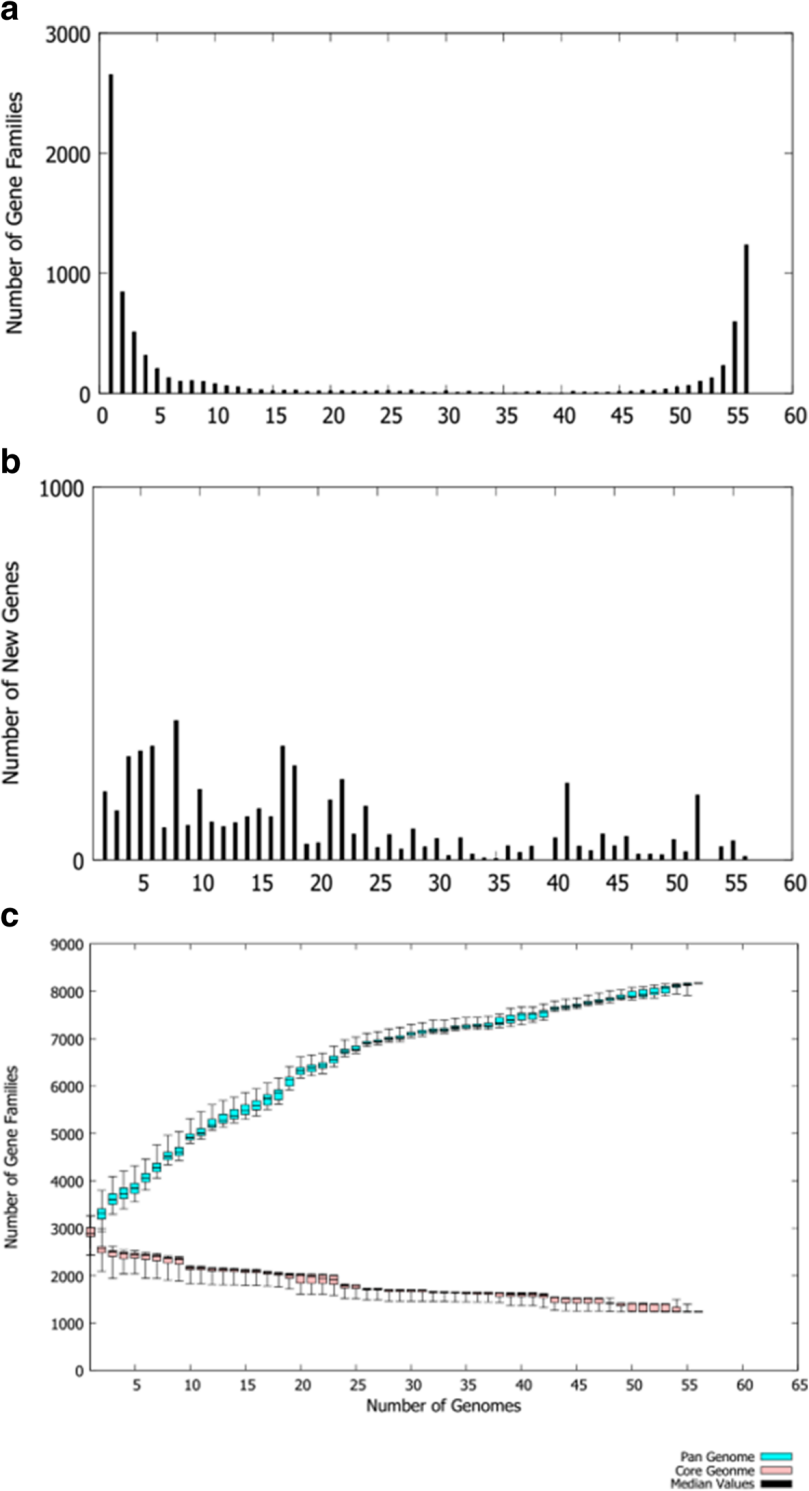
Presence/absence distribution of clusters. **a**. Histogram showing gene family distribution. **b**. Bar chart showing the number of new sequence clusters found in sequentially added *C. perfringens* genomes. **c**. Dot plot showing pan genome and core genome clusters in the *C. perfringens* genomes

The strains sequenced were selected to cover a wide range of spatial and temporal variability. They were isolated from both diseased and healthy chickens at various time periods and from several geographical locations (Table 1). Sequencing statistics can be observed in Table 2. Of the strains examined in this study 38 were poultry strains and 21 were non-poultry strains. The estimated size of the genomes, as predicted from total contig size, ranged from 2,897,393 bp to 3,751,199 bp (Table 2), with an average length of 3,353,566 bp. The pangenome size (i.e. the total number of genes within a group of genomes) was determined to give a measure of the relative complexity within the *C. perfringens* genomes. With the newly sequenced genomes added to those already in the public databases, the pangenome for the strain collection totals 8102 predicted genes, of which 3619 have a predicted function, 2866 are hypothetical conserved protein-coding sequences, which have been found elsewhere, and 1617 are genes of unknown function (novel coding sequences). Between 2611 and 4897 (mean = 3316; s.d. = 509) open reading frames (ORFs) were identified within individual strains. No correlations above 0.03 (Pearson’s R) were observed between the number of ORFs and sequencing method or any of the assembly metrics (number of contigs, total assembly length, or N50) indicating that neither the assembly quality nor the sequencing method affected the analysis. The core genome conserved among all strains consisted of 1192 ORFs, which ranged from 31% (JGS1721) to 43% (SM101) of the total ORFs in a genome. 2454 ORFs were singletons, present in only one strain. An overview of the data generated from 56 strains of *C. perfringens* is presented in Fig. 1.

**Table 1:** Strains analysed in this study

Strain	Toxinotype	*netB+*	Source	Associated Disease	Country	Genome sequence	Clade	Reference	Accession
0522A_28_397	A	–	Human	unknown	USA	+	NP	NCBI	MKXR0000000.1
1207_CPER	A	–	Hospital	unknown	USA	+	NP	[81]	JVYV0000000
2789STDY	A	–	Human	No Disease	–	+	NP	Sanger	CYYX0000000
94–5224	A	+	Chicken	Necrotic enteritis	USA	–	–	This study	^a^
96–7415	A	+	Chicken	Necrotic enteritis	USA	+	3	This study	PJTF00000000
98.78718–2	A	+	Chicken	Necrotic enteritis	DEN	+	1B	[25]	PJTE00000000
ATCC3626	B	–	Lamb	–	–	+	NP	[18]	ABDV0000000
ATCC13124	A	–	Human	Gas Gangrene	–	+	NP	[14]	CP000246
BER-NE33^2^	A	–	Chicken	Necrotic enteritis	AUS	+	3	[25]	PJTD00000000
CP4^1^	A	+	Chicken	Necrotic enteritis	CAN	+	1A	[23]	LIYI0000000
EHE-NE3	A	+	Chicken	Necrotic enteritis	AUS	–	–	[82]	^a^
EHE-NE4	A	+	Chicken	Necrotic enteritis	AUS	–	–	[82]	^a^
EHE-NE5^1^	A	+	Chicken	Necrotic enteritis	AUS	–	–	[82]	^a^
EHE-NE7	A	+	Chicken	Necrotic enteritis	AUS	+	2	[82]	PJTC00000000
EHE-NE18^1^	A	+	Chicken	Necrotic enteritis	AUS	+	1B	[10]	CP025501
EHE-NE20	A	+	Chicken	Necrotic enteritis	AUS	–	–	[10]	^a^
EUR-NE15^1^	A	+	Chicken	Necrotic enteritis	AUS	+	1B	[10]	PJTB00000000
F262	A	–	Cow	Bovine abomastis	CAN	+	NP	[11]	AFES0000000
F4969	A	–	Human	non-food-borne diarrhoea	Europe	+	NP	[18]	ABDX0000000
FC1	A	+	Chicken	Necrotic enteritis	USA	–	–	This study	^a^
FC2	A	+	Chicken	Necrotic enteritis	USA	+	1B	This study	PJTA00000000
FORC_003	A	–	Human	Food poisoning	SKR	+	NP	FORC	CP009557
FORC_025	A	–	Human	Food poisoning	SKR	+	NP	FORC	CP013101
GNP-1	A	+	Chicken	Necrotic enteritis	USA	+	1A	This study	PJSZ00000000
ITX1105-12MP	A	+	Chicken	Necrotic enteritis	USA	+	1A	This study	PJSY00000000
JGS1495	C	–	Pig	Diarrhoea	–	+	NP	[18]	ABDU0000000
JGS1721	D	–	Sheep	Enteritis	–	+	NP	[18]	ABOO0000000
JGS1987	E	–	Cow	Enteritis	–	+	NP	[18]	ABDW0000000
JGS4143^1^	A	+	Chicken	Necrotic enteritis	USA	+	2	[23]	
JJC	A	–	Environment	No Disease	MY	+	NP	[18]	AWRZ0000000
JP55	A	–	Horse	Necrotizing enteritis	CAN	+	NP	[83]	CP010993
JP838	A	–	Dog	Necrotizing enteritis	CAN	+	NP	[83]	CP010994
K473	A	–	Chicken	Necrotic enteritis	AUS	+	3	This study	PJSX00000000
Kendall^1^	A	+	Chicken	Necrotic enteritis	USA	–	–	This study	^a^
Mugdale 5	A	–	Chicken	Healthy	AUS	–	–	[30]	^a^
NAG-NE1^2^	A	–	Chicken	Necrotic enteritis	AUS	+	3	[82]	PJSW00000000
NAG-NE25^2^	A	–	Chicken	Necrotic enteritis	AUS	+	3	[82]	
NAG-NE31^1^	A	+	Chicken	Necrotic enteritis	AUS	+	1A	[25]	PJSV00000000
NCTC 8239	A	–	Human	Food poisoning	UK	+	NP	[18]	ABDY0000000
NobL1^2^	C	–	Chicken	Necrotic enteritis	USA	+	3	This study	PJSU00000000
PBD1^2^	A	–	Chicken	Healthy	AUS	+	4	[30]	PJST00000000
PBS4	A	–	Chicken	Healthy	AUS	–	–	[30]	^a^
PBS5^2^	A	–	Chicken	Healthy	AUS	+	4	[30]	PJSS00000000
PC5^2^	A	–	Chicken	Healthy	AUS	+	4	[30]	PJSR00000000
Pennington	A	+	Chicken	Necrotic enteritis	USA	+	1A	This study	PJSQ00000000
SAF-1	A	–	Chicken	Healthy	AUS	+	3	This study	PJSP00000000
SM101	A	–	Human	Food poisoning	UK	+	NP	[14]	CP000312
11^2^	A	–	Chicken	Healthy	BEL	+	3	[35]	PJSO00000000
13^2^	A	–	Soil	Gas gangrene	–	+	NP	[66]	BA000016
37^1^	A	+	Chicken	Necrotic enteritis	BEL	+	2	[35]	PJSN00000000
48^2^	A	–	Chicken	Necrotic enteritis	BEL	+	3	[35]	PJTN00000000
67^1^	A	+	Chicken	Necrotic enteritis	BEL	+	2	[35]	PJSM00000000
SOM-NE34^1^	A	+	Chicken	Necrotic enteritis	AUS	+	2	[25]	PJSL00000000
SOM-NE35^1^	A	+	Chicken	Necrotic enteritis	AUS	+	2	[25]	PJTM00000000
TAM-NE38	A	+	Chicken	Necrotic enteritis	AUS	+	1A	This study	PJTL00000000
TAM-NE40	A	+	Chicken	Necrotic enteritis	AUS	+	1A	This study	PJSK00000000
SYD-NE41	A	+	Chicken	Necrotic enteritis	AUS	+	1B	This study	PJUV00000000
TAM-NE42	A	+	Chicken	Necrotic enteritis	AUS	+	3	This study	PJSJ00000000
TAM-NE43	A	+	Chicken	Necrotic enteritis	AUS	+	3	This study	PJTK00000000
TAM-NE46	A	+	Chicken	Necrotic enteritis	AUS	+	1A	This study	PJSI00000000
TAM-NE47	A	+	Chicken	Necrotic enteritis	AUS	–	1A	This study	^a^
T3381	A	–	Chicken	Necrotic enteritis	AUS	+	3	This study	PJTJ00000000
UDE 95–6812	A	+	Chicken	Necrotic enteritis	USA	+	1A	This study	PJTI00000000
W1319	A	–	Chicken	Necrotic enteritis	AUS	+	3	This study	PJSH00000000
WAL-14572	A	–	Human	No Disease	–	+	NP	[18]	ADLP0000000
Warren^1^	A	+	Chicken	Necrotic enteritis	USA	+	1A	This study	PJTH00000000
WER-NE36^1^	A	+	Chicken	Necrotic enteritis	AUS	+	2	[84]	PJTG00000000

^1^Isolate has been tested in a disease induction model and demonstrated to be virulent

^2^Isolate has been tested in a disease induction model and demonstrated to be avirulent

^a^These isolates were not sequenced. They were only used for PFGE analysis

**Table 2:** Sequencing statistics

Strain	Chromosomal Contigs	Total bp	Max contig length (bp)	Min contig length (bp)	N50	Potential genes (Chromosomal genes)	Accessory genes	Unique genes	Exclusively absent genes
0522A	31	3,417,203	799,085	1716	250,061	3165	1680	81	0
1207_CPER	95	3,206,961	279,830	204	85,634	2818	1511	28	0
2789STDY	48	3,321,076	2,127,284	377	903,119	2979	1566	32	0
96–7415	49	3,388,438	797,628	1527	244,647	3932	1651	86	0
98.78718–2	36	3,417,550	410,887	2042	196,455	3963	1772	35	3
ATCC3626	64	3,585,098	299,576	1616	91,764	4159	1760	83	5
ATCC13124	1	3,256,683	3,256,683	3,256,683	3,256,683	3719	1536	38	11
BER-NE33	30	3,349,933	3,345,877	4056	3,345,877	4107	1558	38	19
CP4	96	3,590,658	289,484	1282	83,876	3371	1500	211	42
EHE-NE7	11	3,257,283	2,079,954	4153	2,079,954	4368	1597	0	0
EHE-NE18	1	3, 474,200	3, 474,200	3, 474,200	3, 474,200	3965	1783	0	0
EUR-NE15	11	3,378,967	2,213,684	2319	2,213,684	4672	1755	21	4
F262	1	3,333,039	3,333,039	3,333,039	3,333,039	3793	1667	46	0
F4969	61	3,420,663	365,849	1632	113,358	3997	1605	85	8
FC2	24	3,297,593	986,908	4358	966,190	3867	1615	27	0
FORC_003	1	3,338,532	3,338,532	3,338,532	3,338,532	3060	1603	17	0
FORC_025	1	3,343,822	3,343,822	3,343,822	3,343,822	3038	1632	43	0
GNP-1	26	3,522,496	1,064003	2009	363,806	4185	1905	11	0
ITX1105-12MP	259	3,277,088	115,260	1547	22,062	4424	1866	0	0
JGS1495	59	3,412,610	383,044	1664	117,588	4003	1572	41	5
JGS1721	104	3,608,664	192,053	1519	80,514	4331	1660	50	8
JGS1987	72	3,751,199	270,422	1671	96,857	4397	1772	255	5
JGS4143	318	3,658,663	3,658,663	3,658,663	3,658,663	4124	1657	183	0
JJC	54	3,203,273	445,718	1595	98,246	3724	1533	24	1
JP55	1	3,347,300	3,347,300	3,347,300	3,347,300	3266	1618	21	0
JP838	1	3,530,414	3,530,414	3,530,414	3,530,414	3807	1734	114	4
K473	46	3,328,345	724,340	2307	158,573	6564	1316	130	91
NAG-NE1	106	3,315,837	2,659,148	2002	2,659,148	4994	1434	92	43
NAG-NE25	247	3,408,683	3,408,683	3,408,683	3,408,683	3953	1707	50	2
NAG-NE31	72	3371,019	1,031,391	2265	683,309	5198	1684	26	2
NCTC 8239	45	3,313,110	524,606	1602	146,935	3784	1520	33	6
NobL1	10	3,574,575	3,403,922	2168	3,403,922	4605	1679	53	5
PBD1	13	3,176,182	1,454,083	2985	572,872	5498	1509	44	40
PBS5	10	3,110,163	2,317,119	2322	2,317,119	4377	1578	18	7
PC5	11	3,103,348	2,196,352	2335	2,196,352	4284	1602	24	4
Pennington	35	3,501,064	1,037,768	2204	228,190	4158	1903	4	0
SAF-1	80	3,356,947	3,354,395	2552	3,354,395	3934	1605	37	0
SM101	1	2,897,393	2,897,393	2,897,393	2,897,393	3299	1136	62	93
Strain 11	35	3,197,244	515,326	4530	206,922	3662	1523	15	0
Strain 13	1	3,031,430	3,031,430	3,031,430	3,031,430	3438	1642	15	7
Strain 37	36	3,255,773	461,756	1501	148,687	3764	1593	11	0
Strain 48	33	3,250,386	670,855	1575	245,313	3738	1552	52	0
Strain 67	34	3,268,421	451,598	1501	243,534	3794	1626	21	0
SOM-NE34	25	3,418,437	3,355,569	62,868	3,355,569	3952	1720	1	0
SOM-NE35	35	3,364,015	1,180,705	2351	878,365	4722	1748	36	1
TAM-NE38	13	3,447,589	3,395,713	2411	3,395,713	4063	1801	0	0
TAM-NE40	15	3,452,011	3,395,271	2201	3,395,271	4086	1797	2	1
SYD-NE41	15	3,542,115	3,502,074	2002	3,502,074	4557	1790	36	5
TAM-NE42	96	3,355,605	3,353,147	2458	3,353,147	4015	1613	17	0
TAM-NE43	18	3,272,035	1,846,259	4695	1,846,259	3780	1596	27	0
TAM-NE46	33	3371,805	492,250	1589	278,745	4003	1791	0	0
T3381	67	3,335,304	352,891	2762	119,157	7258	1220	125	161
UDE 95–6812	25	3,366,442	1,428,911	2009	381,265	4004	1766	0	0
W1319	11	3,236,902	2,023,168	2290	2,023,168	4667	1568	35	9
WAL-14572	26	3,403,365	618,435	2272	307,728	3928	1721	50	1
Warren	14	3,561,701	3,425,489	2387	3,425,489	4408	1866	9	1
WER-NE36	113	3,283,756	3,281,264	2492	3,281,264	4536	1602	12	3

**Fig. 2:**
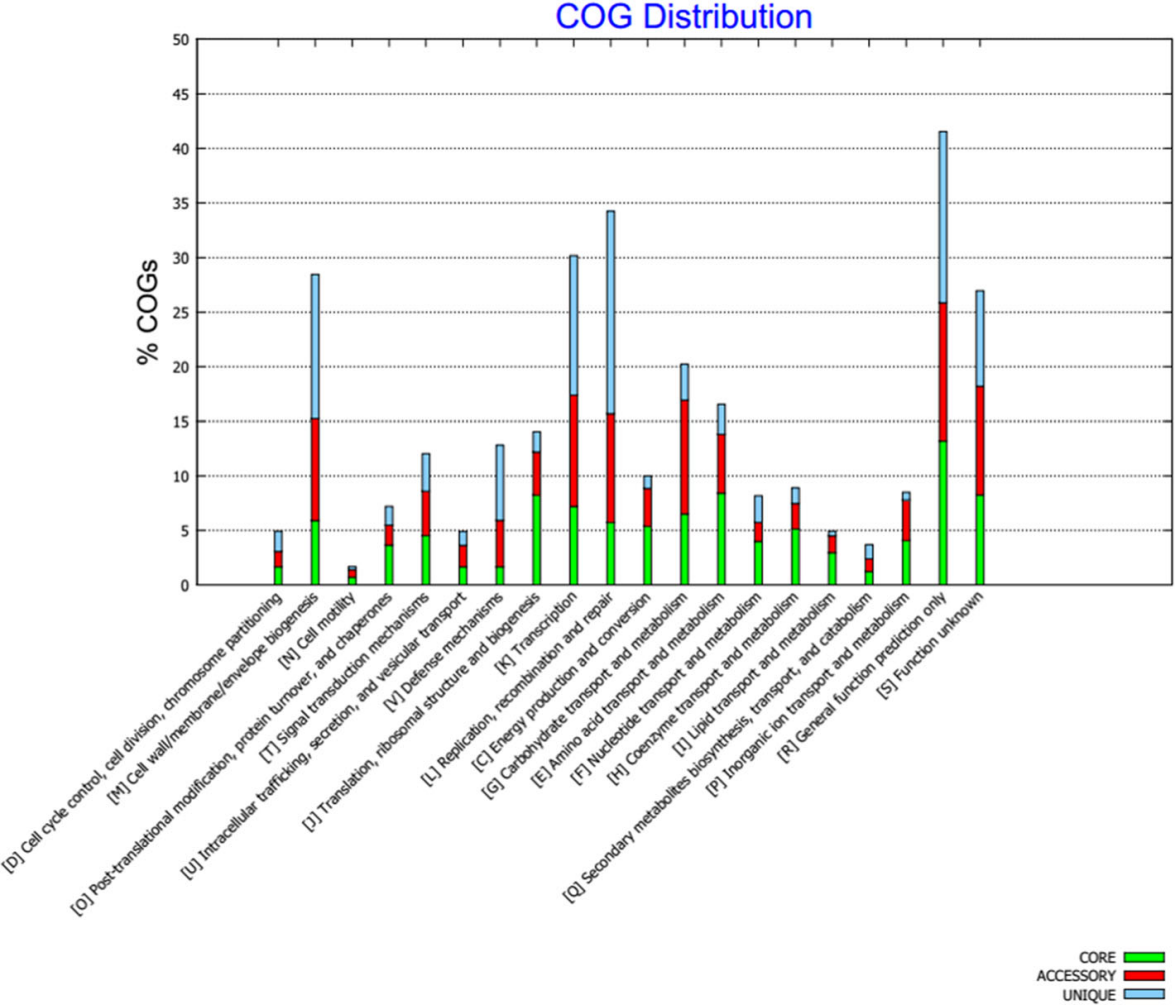
COG functional analysis of pan-genome. The histogram shows the predicted functionality of proteins assigned to core, accessory and singleton clusters

To investigate the functionality of the pan-genome, a blast search was performed from representative sequences of each protein cluster against the COG database. As shown in Fig. 2, the functional categories of the proteins were assigned to core, accessory and singleton classes. The accessory and singleton genes encode proteins with a very large amount of functional diversity in comparison to the core proteins, suggesting that a wide range of functional variation is possible between strains based on the accessory genes that they carry.

### Evolutionary relationships of isolates revealed by pan-genome analysis

A pan genome binary matrix was produced from the gene clusters, which were calculated using the UCLUST algorithm [29], and the presence or absence of a cluster was assigned to each strain. We found 8002 unique clusters, corresponding to the total gene content of the pan-genome. An average of 4039 clusters was assigned to each strain.

**Fig. 3:**
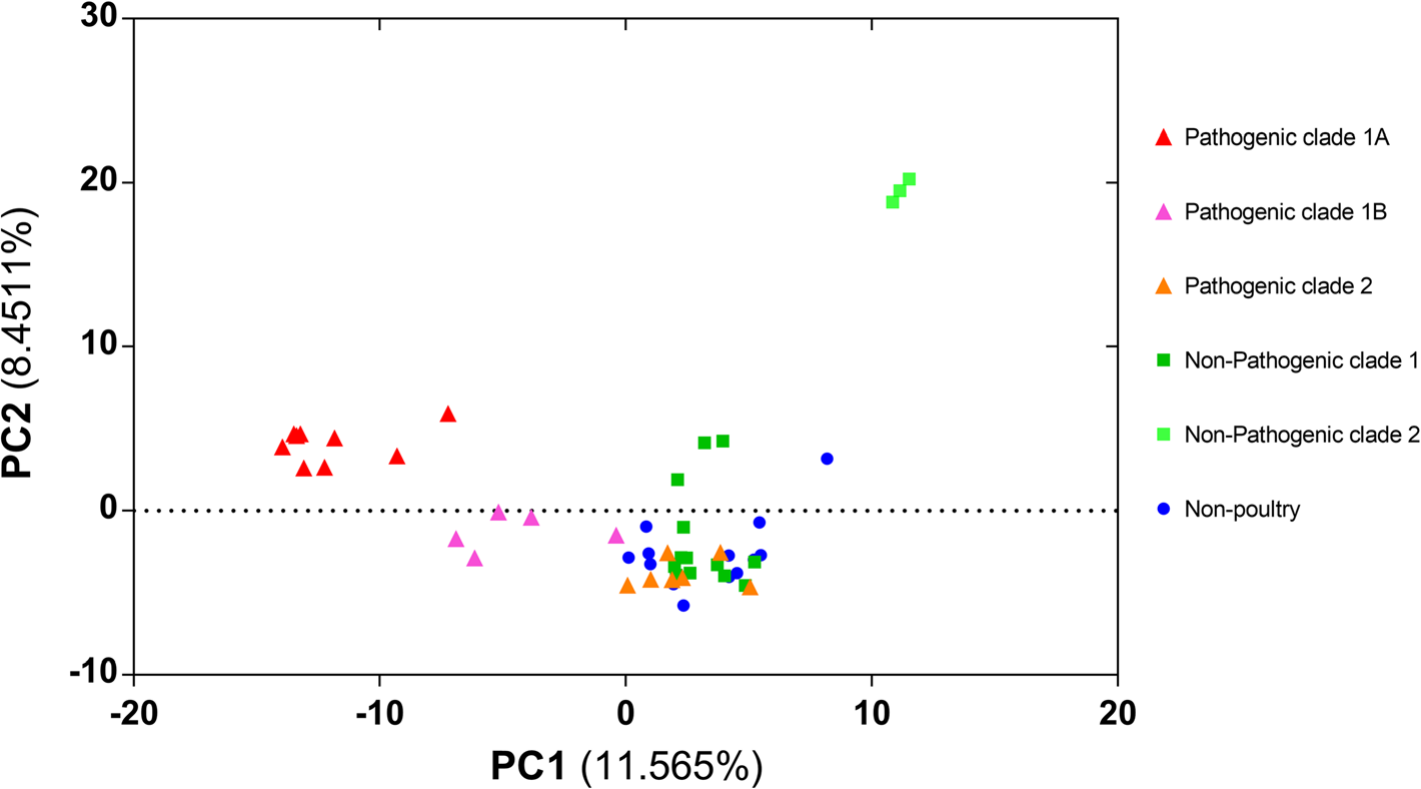
PCA on the pan-matrix. Each dot marks a genome in the first two principal components of the pan-matrix**.** Strains are coloured by clade designation and disease-causing capability. Shapes represent; triangle, *netB* positive from diseased birds; squares, *netB* negative poultry isolates; circle, non-poultry *netB* negative isolate

The pan genome binary matrix was used as input in a principal component analysis (PCA) in XLSTAT to reveal the dominant differences between strains. Major differences between isolates, with several distinct clusters of isolates, were defined (Fig. 3). Two clusters of non-pathogenic chicken isolates (dark green and light green) were seen, one being clustered with non-poultry strains in the bottom right of the plot and a second very different group at the top of the PCA plot (Fig. 3). Most non-poultry strains clustered closely together, while the pathogenic chicken strains fell into three distinct clusters (dark red, light red and orange) (Fig. 3). One of the pathogenic chicken clusters is closely related to the non-poultry strains and to one of the non-pathogenic chicken clusters (Fig. 3).

**Fig. 4:**
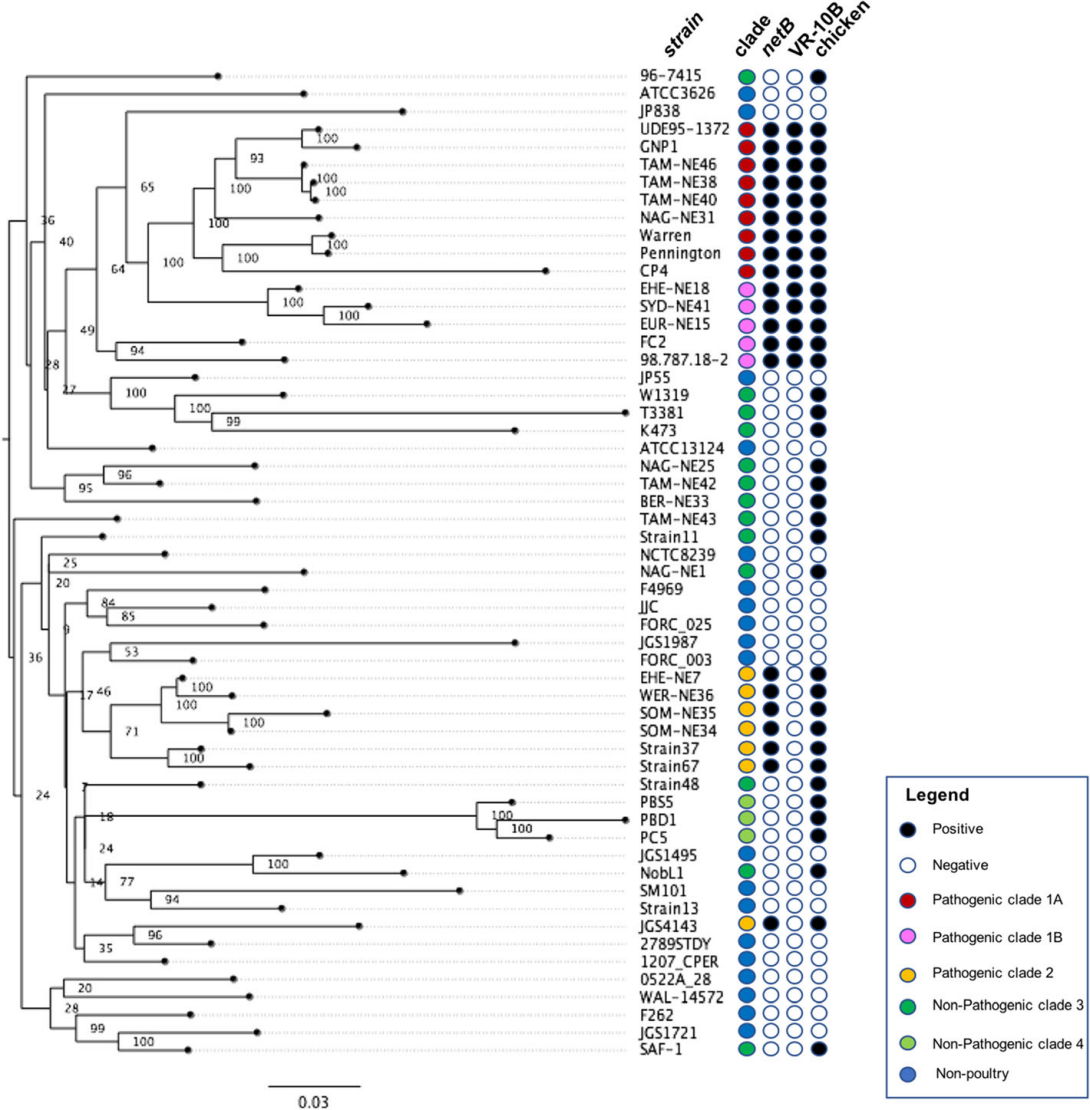
Total gene content phylogeny. Total gene content phylogeny. Tree of maximum likelihood based on total gene content of the chromosome of various *C. perfringens* strains. Red, Pink represent pathogenic strains of *C. perfringens* clades 1A, and 1B respectively, while orange represent pathogenic clade 2. Dark Green and light green represent non-pathogenic strains in clades 3 and 4 respectively. Black, positive; white, negative

Since the PCA indicated some clustering that correlated with disease capability (Fig. 3), the genomes were investigated by producing a pan-genome tree of maximum likelihood to compare the total gene content of the strains. The pangenome tree showed two distinct clades of pathogenic chicken strains (Fig. 4). One clade of pathogenic strains (orange) has close relationships to the majority of non-pathogenic poultry and non-poultry strains, supporting the PCA observations. The other clade of pathogenic chicken strains shares a monophyletic branch on the tree (pink and red), however, there appears to be some divergence between strains within this group (Fig. 4). Following examination of the tree structure, pathogenic isolates were assigned to two major clades, pathogenic clades 1 and 2. Pathogenic clade 1 was split into sub-clades to account for both PCA diversity and divergence in the tree structure; two sub-clades were defined; pathogenic subclade 1A (red) and subclade 1B (pink). The other pathogenic poultry clade was designated pathogenic clade 2 – orange (Fig. 4). The non-pathogenic strains also fell into two clusters. The large clade, closely related to non-poultry strains, consisted of strains from both healthy and diseased poultry, but use of these strains in our necrotic enteritis disease induction model provided evidence that these strains were not capable of causing disease; this clade was designated non-pathogenic clade 3. A highly divergent group of strains formed non-pathogenic clade 4 (Fig. 4), and consisted of strains isolated from healthy chickens that were not capable of causing disease in a chicken necrotic enteritis infection model. Clades showed no correlation to country of origin or time of isolation, with the notable exception of non-pathogenic clade 4, which consists of only Australian isolates isolated in 2005.

### Variable chromosomal regions identified in *C. perfringens* poultry isolates

**Fig. 5:**
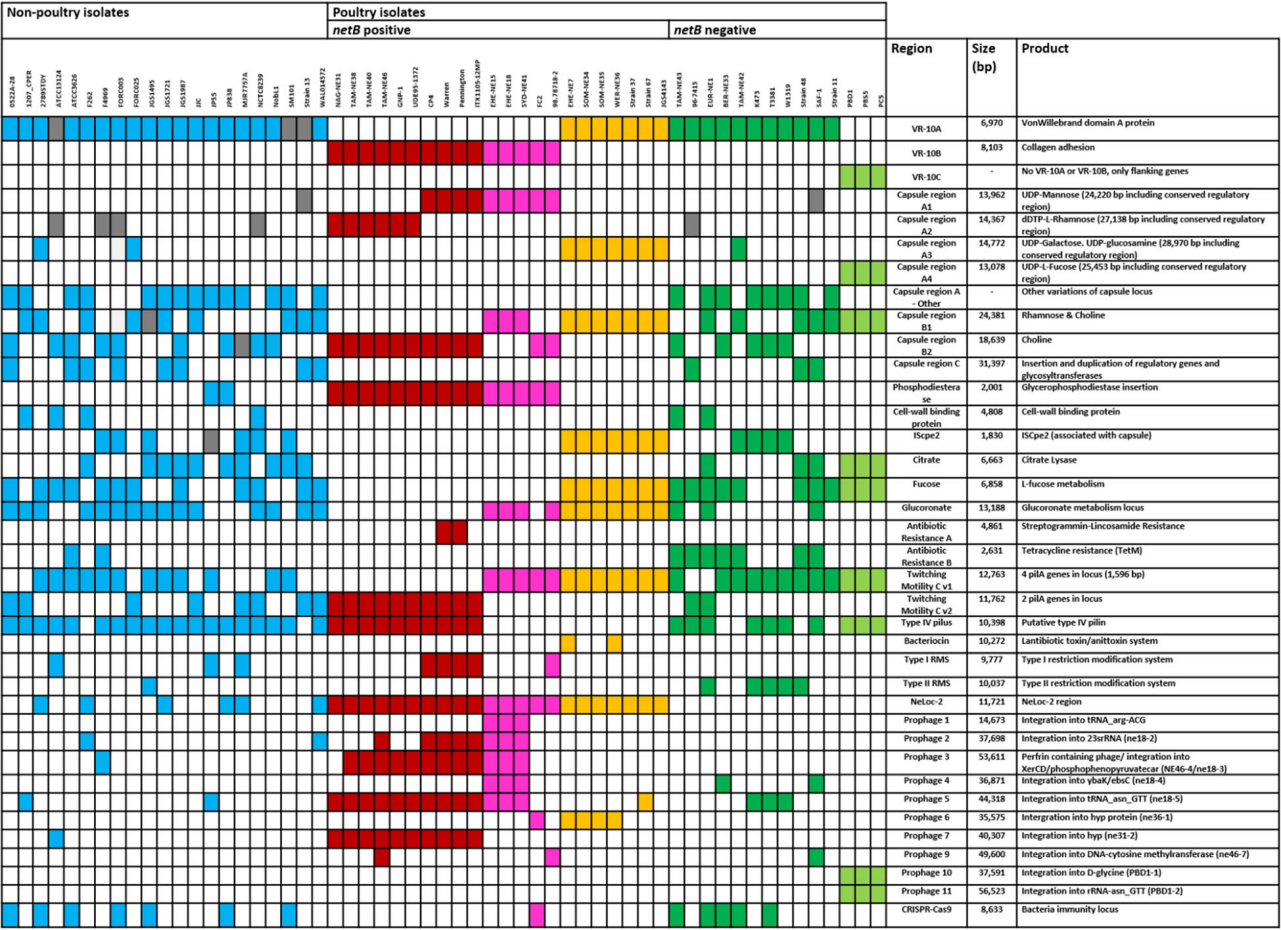
Presence of variable regions. Heatmap showing the presence and absence of variable chromosomal loci among 38 poultry strains and 21 non-poultry strains. Each column represents a different strain and each row represents a different chromosomal locus. Coloured sections represent a positive hit for that strain, grey represents a partial match (not entire locus but a single gene) and white is no match. Colours represent clade designation of a strains with pathogenic clade 1A (red), pathogenic clade 1B (pink) and pathogenic clade 2 (orange) while non-pathogenic clade 1 (dark green) and non-pathogenic clade 2 (light green). Non-poultry strains are coloured blue

To further examine the basis of strain separation and divergence, the chromosomal content of each strain was investigated and compared. Several hundred genes were variably present in each strain. A binary matrix was used to determine which clusters were exclusively present or absent within and between the designated clades. Many of the genes responsible for the variation between strains were grouped within specific genomic loci or regions. These regions were mapped against the chromosome of each strain to establish their relative location within the genomes. These variable regions encoded proteins with putative and known roles in colonisation, phage integration, capsule polysaccharide biogenesis, motility, antimicrobial activity, and alternative metabolism pathways. Many of these regions encoded genes that were associated with mobile genetic elements (Fig. 5) such as transposase genes, recombinase genes and IS elements, or were flanked by highly conserved regions shared by all strains.

#### Adhesion-associated locus

**Fig. 6:**
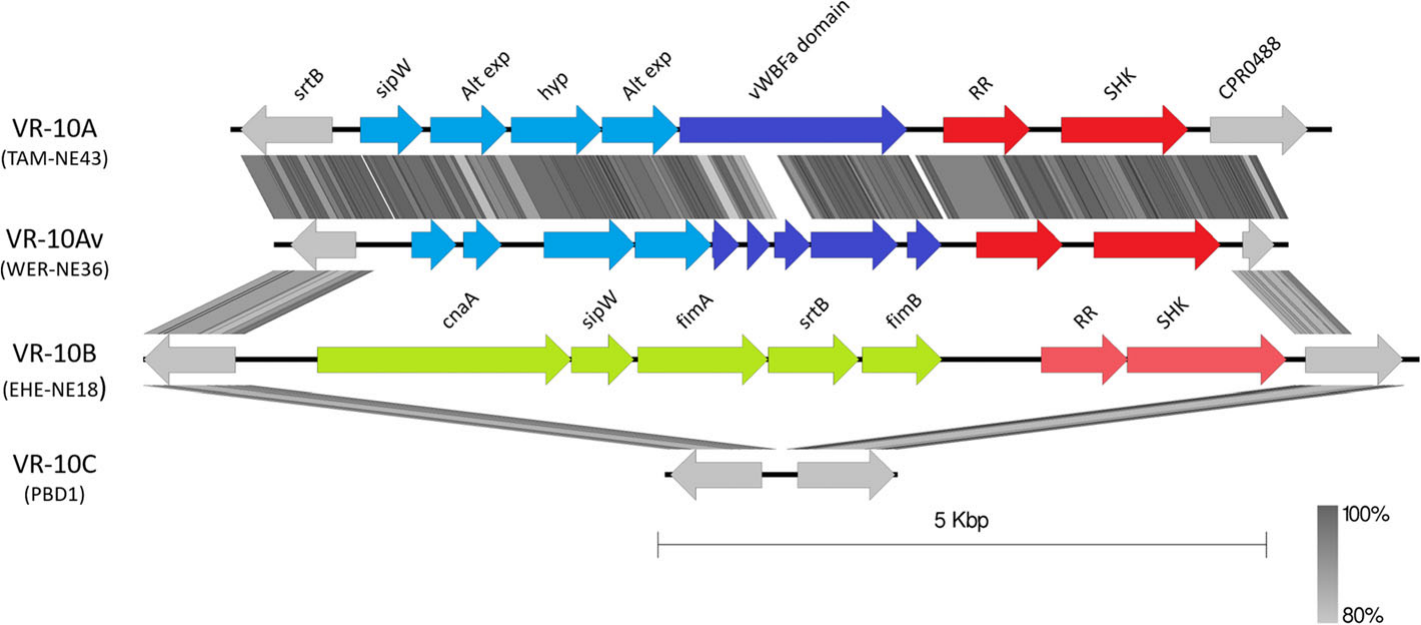
Adhesion locus variations. Genetic map showing sequence alignment of the different adhesion associated loci. Alignment is shown for four strains TAM-NE43, WER-NE36, EHE-NE18 and PBD1. TAM-NE43 has the intact VR-10A (blue) variant, WER-NE36 shows a highly deleted variation of VR-10A, VR-10B is represented by EHE-NE18 (green) [30] and PBD1 represents the VR-10C variant. Flanking genes of the locus sortase (*srtB*), and CPR0488 are coloured grey, and the two variants of the response regulator and sensor histidine kinase (SKH) are coloured red. Abbreviations: Alt exp.; alternative export protein, sipW; signal peptidase, hyp; hypothetical proteins, RR; response regulator, SHK; sensor histidine kinase, vWBFa; vonWillebrand factor A-like protein

**Fig. 7:**
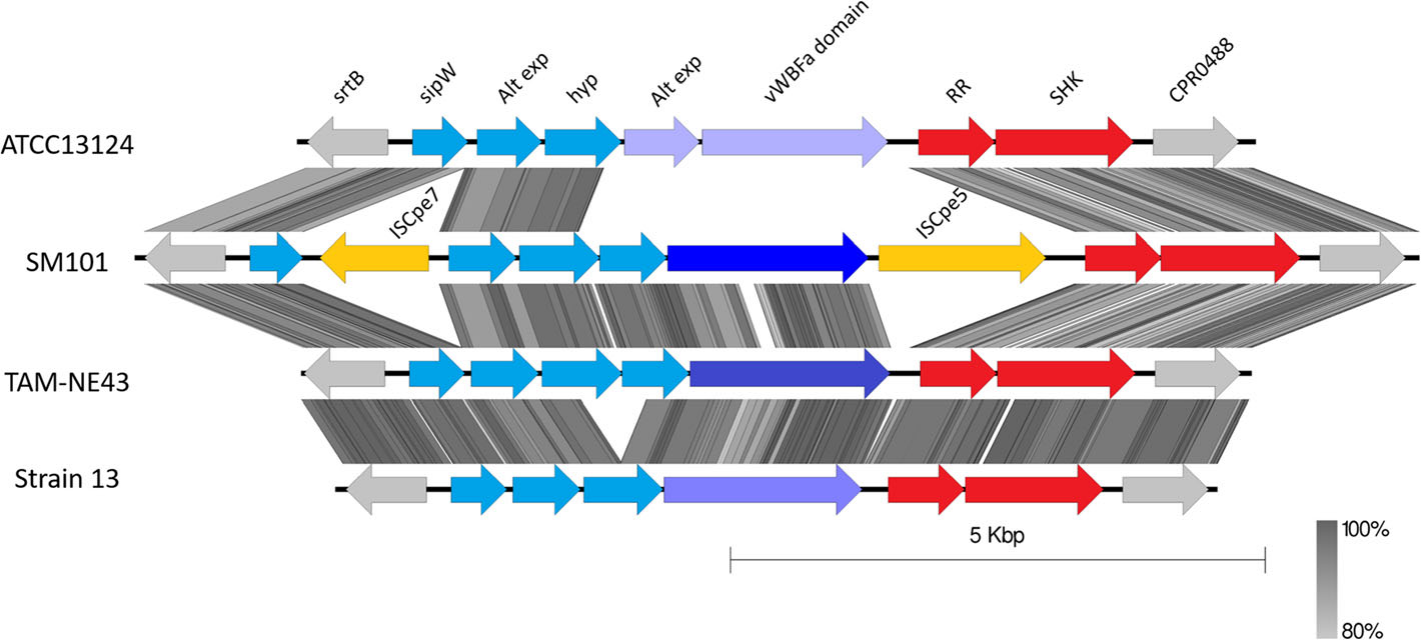
VR-10A variations. A genetic map showing sequence alignments of the different VR-10A adhesion loci. Alignment is shown for four strains: ATCC13124, SM101, TAM-NE43 and 13

One region that varies between the pathogenic clades was an adhesion-associated locus (Fig. 6). This locus has three major variants and is located between two highly conserved genes present in all strains, which encode a sortase B enzyme and a CPR048-like protein. The first two variants have been previously described as VR-10A, encoding a vonWillebrand domain A protein, and VR-10B, encoding a collagen adhesion fimbriae-like protein [23, 30, 31]. Our work has provided further insights into this region, specifically; that a third variant, VR-10C, contains neither of the two previously described loci but instead only encodes the flanking genes (only in strains in non-pathogenic clade 4). VR-10B is exclusively found in pathogenic clade 1 and is highly conserved. VR-10A is the most common variant present in all other strains. VR-10A is, however, highly variable, with many mutations detected in the poultry strains, which may suggest gene decay due to its inessential function (Fig. 6). Major variations of VR-10A were observed in the non-poultry strains SM101, ATCC13124 and 13, each having a unique variant of the locus (Fig. 7).

#### Capsular polysaccharide synthesis locus (CpCap)

**Fig. 8:**
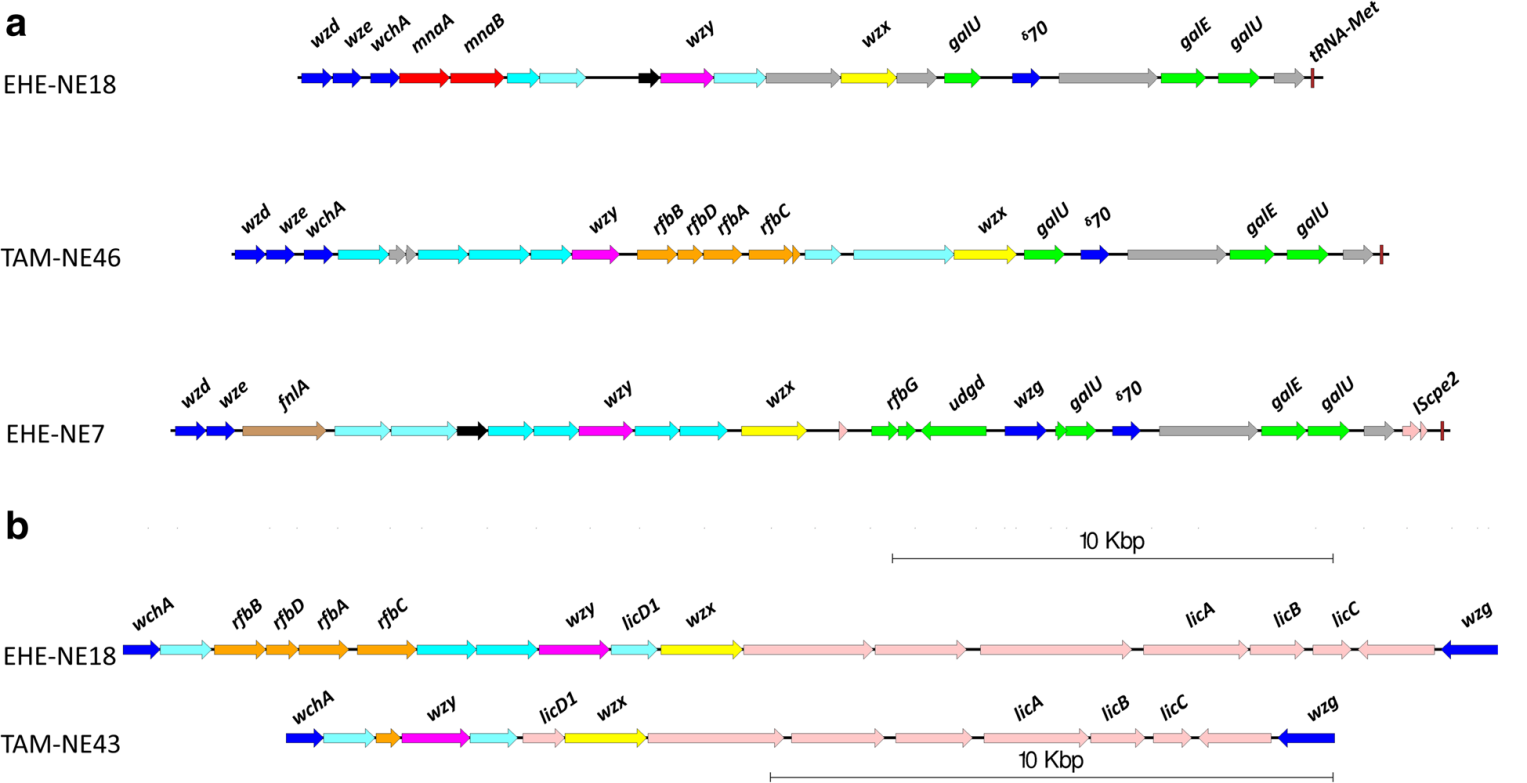
Capsule polysaccharide synthesis locus variations. Genetic map comparing the three capsule polysaccharide synthesis (CpCap) loci in pathogenic strains of *C. perfringens.* Representative regions from EHE-NE18 (mannose), TAM-NE46 (rhamnose) and EHE-NE7 (glucosamine) and the CpCPSL-B variants. **a**. Cds regions are colored as follows: Dark Blue – regulation, light blue – glycosyltransferase, red - UDP-mannose, orange – dTDP-rhamnose, Brown – UDP-L-fucose, Green – UDP-glucosamine/UDP-galactose, Dark pink – polysaccharide polymerase, yellow- polysaccharide transporter/flippase, Black – acetyltransferase, grey – hypothetical/unknown, Light Pink – mobile genetic elements and light green UDP-glycerol. **b**. As above but also Light Pink – phosphocholine

Directly upstream of the adhesion-associated locus is a large capsular polysaccharide synthesis locus (CpCap). One of the differentiating chromosomal regions between pathogenic clades is the variation within capsular polysaccharide synthesis genes. There are two major chromosomal regions predicted to play a role in capsular polysaccharide biosynthesis [14]. These regions vary in composition between strains, most likely resulting in different capsular polysaccharide structure (Fig. 8a and b).

In all strains the first large capsule region, designated “Cap A” (Fig. 8a), encodes genes required for the modulation and biosynthesis of the capsule, including homologs of the *cpsCDE* (*wze, wzd, wchA*) genes at the 5′ region and genes encoding UDP-galactose-4-epimerase (*galE*), two copies of UTP-glucose-phosphate uridyltransferase (*galU*), and an RNA polymerase sigma factor 70 at the 3′ end of the locus. However, individual strains carry a diverse set of glycosyltransferases and UDP-sugar synthesis genes that encode the production of one or a combination of UDP-fucose, UDP-mannose, UDP-glycerol and/or dTDP-rhamnose, to be assembled as a polysaccharide capsule. Strains also encode a varied set of capsule polymerases (Wzy-like) and flippases (Wzx-like), suggesting specificity for the encoded UDP-sugar pathway. In some strains, a duplication of the modulation region *cpsCD* is observed upstream of the first locus, resulting in a much larger capsule polysaccharide synthesis locus, however, this duplication has not been observed in pathogenic poultry strains.

The second capsule region, designated “Cap B”, is more conserved than capsule region A, in most strains the presence of this locus is correlated with capsule locus A. In all strains the second locus encodes another undecaprenyl-phosphate galactose phosphotransferase (CpsE/WchA) at the 5′ end of the locus and a homolog of the capsule regulator Wzg/CpsA (LytR response regulator) at the 3′ end (Fig. 8b). In all strains, this region contains a set of genes encoding phosphocholine biosynthesis and export and, like region A, encodes a capsule polymerase (Wzy-like), a flippase (Wzx-like) and a set of glycosyltransferases. Most strains that do not contain proteins required for dTDP-rhamnose synthesis in the first capsule region encode these proteins in the second region, except for some strains in pathogenic clade 2, which do not encode any dTDP-rhamnose biosynthesis genes.

Pathogenic clade 1 strains encode proteins required for the production of UDP-mannose, UDP-rhamnose and phosphocholine within these capsule regions. However, some strains within pathogenic clade 1 lack the UDP-mannose biosynthesis genes. Pathogenic clade 2 and some strains from non-pathogenic clade 3 appear to encode a simple capsule type, as they have neither UDP-mannose or UDP-rhamnose coding regions. In these strains, the presence of a glucose-dehydrogenase-encoding gene predicts that the pathogenic clade 3 strains encode a galactose/glucosamine capsule.

Many of the non-pathogenic clade 3 strains encode the most diverse capsules, with several different types observed. Strain 11, for example, encodes proteins in the capsule region A that are predicted to make fucose additions to the capsule while strains SAF-1 and 96–7415 have a duplication of the modulation genes in the capsule locus A as well as encoding glycerol modification proteins. Strains within pathogenic clade 2 and the non-pathogenic clades that do not encode a duplication of the modulation region only encode *cpsE/WchA* in the capsule region; instead, in region A they encode a UDP-N-acetyl-glucosamine 4,6-dehydratase (via the *pseB/fnlA* genes).

Overall, pathogenic poultry strains only appear to encode three different capsule polysaccharide synthesis loci while non-pathogenic strains and non-chicken strains have highly diverse capsule types.

#### NEloc-2 – Chromosomal necrotic enteritis-associated locus

NEloc-2, previously described by Lepp [22], contains 11 ORFs encoding proteins with various functions, including sigma factors, chitin synthases and CotH-like proteins. This locus was previously found to be highly prevalent in NE-causing *C. perfringens* strains, but in this study, we show that it also is present in non-chicken strains, with the locus observed in JGS1721, F262, WAL-14527, MUR7757A, and JP838. We found that all necrotic enteritis-causing strains carried the NEloc-2 region. Several non-pathogenic strains from healthy and diseased birds, including strain 11, BER-NE33, NAG-NE1 and TAM-NE42, did not encode this locus. In some strains, the NEloc-2 region of the chromosome is replaced by a gene predicted to encode a flavin adenine dinucleotide-binding protein*.* This study further confirms the association of NEloc-2 with necrotic enteritis causing *C. perfringens* isolates.

#### Prophage-like regions

A large amount of the chromosomal variation between strains results from prophage-like integration. The prophage-like regions are diverse in genomic content and vary considerably between strains. In this strain collection, there are approximately 16 unique prophage-like integrations (predicted to be intact by PHAST) in the various chromosomes (Fig. 5). Further prophage diversity was examined by the presence of partial fragments of what are presumed to be remnants of old integration events.

In some clades, the presence of prophage regions on the chromosome is less prominent (pathogenic clade 2 and non-pathogenic strains typically have one or two prophage elements), while in pathogenic clade 1, prophage elements are highly prevalent with multiple prophage regions characterised in each strain. Some of these prophages are co-linear, localised in the same region of the chromosome, with similar insertion sites. Some of the prophage regions are shared by otherwise chromosomally distinct strains. Most of the prophage integrations are distinct from previously characterised *C. perfringens* phage. One strain within pathogenic clade 1, FC2, encodes one phage associated region and is closer to pathogenic clade 2, which also typically contain a single prophage element; it therefore appears that FC2 may be more divergent from the other pathogenic clade 1 strains. One prophage element (Prophage-II) is uniquely associated with non-pathogenic clade 4, which includes strain PBD1. This prophage region is highly distinct from all the other prophage regions showing very little similarity to those in other strains. This region is a major driver for the clear separation observed between strains in non-pathogenic clade 4 and all other isolates. There are also many phage fragments (incomplete phage regions) usually present as singletons, which also contribute to strain diversity.

#### Inhibitory molecules

A chromosomal region encoding a putative antibiotic system/bacteriocin locus was found in some strains within pathogenic clade 2 (WER-NE36, EHE-NE7, SOM-NE34 and SOM-NE35). This locus contains genes predicted to encode two cytolysin-like bacteriocins (ClyM), an immunity protein, several membrane associated proteins and a cytolysin transport protein (Fig. 5). If functional, this locus could provide a selective advantage for these strains over other pathogenic strains within an infected host or within a common niche.

A secondary bacteriocin gene is highly prevalent in necrotic enteritis strains. Previously described as *cpp* (perfrin) [32], this bacteriocin-encoding gene is located within a variable phage-related locus only in some pathogenic necrotic enteritis causing strains. The gene is in several strains from pathogenic clade 1 but no strains in pathogenic clade 2 or any of the non-chicken or non-pathogenic chicken isolates (Fig. 5). Examples of strains that encode *cpp* are those with a phage-related locus with high identity to the EHE-NE18-prophage-III, including Pennington, Warren, GNP-1, SYD-NE41, TAM-NE38 and several others. It is important to note that some strains within pathogenic clade 1, including 98.78718–2 and FC2, do not encode the entire phage region and therefore do not contain the *cpp* gene. It has been shown that *cpp* plays an important role in the inhibition of *C. perfringens* strains [32] and therefore prophage regions are regarded as an important factor for virulence.

This analysis also discovered significant genome variability between strains with predicted functions in metabolism (fucose, citrate and glucoronate), motility (type IV pili), and bacterial immunity (CRISPR and type I restriction modification system). These regions were not observed to be strictly associated with pathogenesis in necrotic enteritis, but rather sporadically present across strains from varied hosts and disease-causing capability. Finally, there are also many singleton ORFs that contribute to the variation seen in the chromosomes of *C. perfringens* strains. The functional role of proteins encoded by these single genes is difficult to determine, but is likely to contribute or influence different metabolic pathways and virulence factors within the strains.

### Plasmid carriage and clade designation

Sequence analysis in this study also provided information about the carriage of plasmids by each isolate. In the earlier analysis plasmid sequences were removed to focus on the chromosomal variation. However, a separate examination of the sequence data for known and unknown plasmids showed that all strains in pathogenic clades 1 and 2 carried *netB* and *cpb2*-encoding plasmids. All strains in pathogenic clade 1 carried a tetracycline resistance plasmid, like the well-characterised prototype plasmid pCW3. Only pathogenic sub-clade 1A strains carried a *tpeL*-encoding plasmid. By contrast, all non-pathogenic poultry strains isolated from healthy birds (SAF-1, strain 11, PBD1, PC5 and PBS5), and a single non-pathogenic strain isolated from a diseased bird (TAM-NE43), did not contain any plasmids. Most of the strains in non-pathogenic clade 3 contained a single plasmid which encoded both tetracycline resistance and the beta2 toxin, with similar composition to the published sequence pFORC03 (Accession Number: NZ_CP009558).

### SNP analysis

**Fig. 9:**
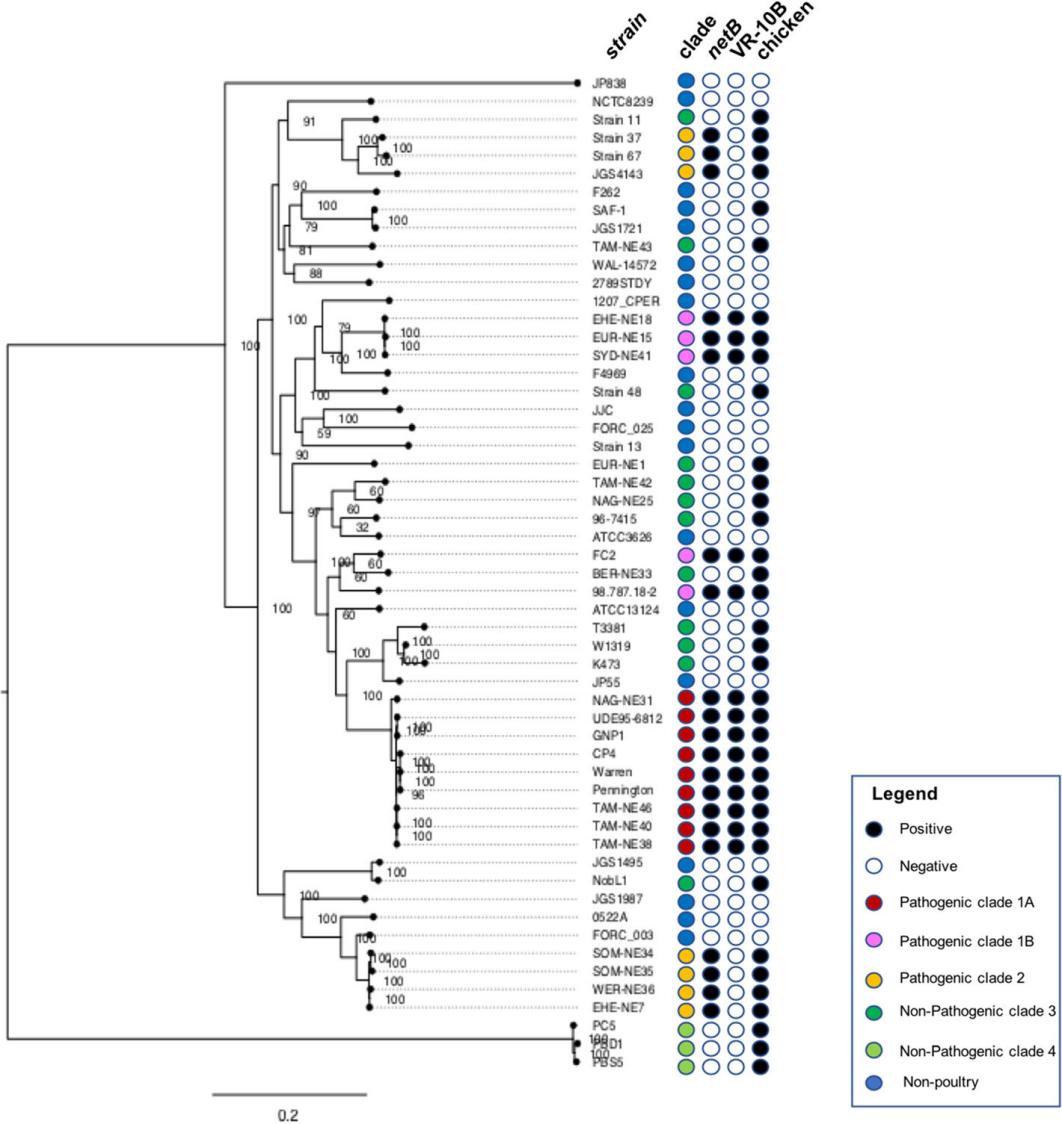
Core genome phylogeny. Tree of maximum likelihood based on single nucleotide polymorphisms in the core chromosome of *C. perfringens*. Dark red and light red squares represent Pathogenic strains of *C. perfringens* clades 1 2 respectively while green triangles and diamonds represent non-pathogenic strains in clades 3 and 4 respectively. Black; positive, white; negative

The core genome (genes shared by all strains) consists of 1192 genes. Analysis of these genes using HARVEST tools v1.2 and Snippy v3.2 with ATCC13124 as the reference strain identified 119,657 core SNPs from 295,709 variant sites. The SNP occurrence in each strain was used to produce a relatedness tree of maximum likelihood (Fig. 9) using RAxML v8.0 general time reversible model with 1000 bootstraps [33]. A comparison of the SNP tree to the Pan-genome tree revealed distinct differences and similarities in the evolutionary relationships calculated using these different methods. The SNP tree indicates that the pathogenic poultry isolates are more widely dispersed than indicated by the pan-genome analysis. Isolates designated as pathogenic clade 1 and pathogenic clade 2 no longer cluster together, but rather are separated into multiple groups, suggesting that the core genome does not closely reflect the gene diversity seen in the pan-genome analysis. Some strains do, however, cluster closely together suggesting that they are highly related and possibly clonal, as clustering is conserved in both core gene SNPs and the pan-genome content. Overall, core genome phylogeny does not match disease causing capability.

### PFGE fingerprinting

**Fig. 10:**
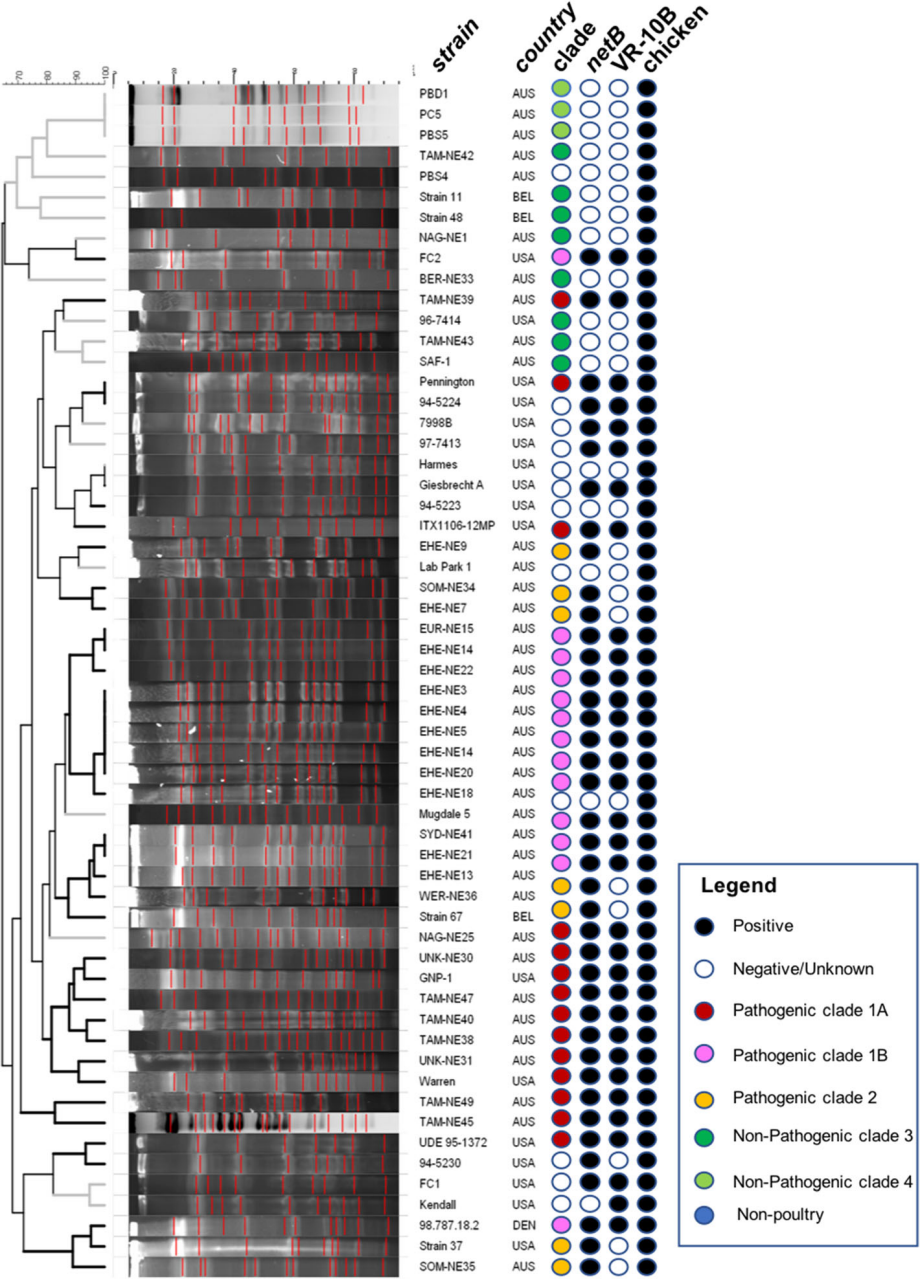
PFGE. Tree of PFGE fingerprints of various *C. perfringens* strains. Colours are as follows; white; negative, black; positive, for features including *netB*, VR-10A and isolation from chicken. Colours referring to clade designation are as follows; blue; Non-poultry, Green (dark and light); Non-pathogenic clade 3 and 4 respectively, Pink; pathogenic clade 1B, Red; pathogenic clade 1A, Orange; pathogenic clade 2

Pulsed-field gel electrophoresis (PFGE) was also used to examine the phylogenetic relationship between strains and to determine if this method could be used to categorize strains into the clades identified by whole genome sequencing. A chromosome digest followed by PFGE analysis revealed that among pathogenic necrotic enteritis-causing strains, three distinctly different PFGE patterns were observed. A dendrogram generated from the PFGE profiles placed all the pathogenic strains into similar groupings as that defined by the chromosome sequence comparison, demonstrating that PFGE gives a good representation of the pan-genome variation that has been observed within the *C. perfringens* strain collection examined here (Fig. 10). Several strains, including EHE-NE3, EHE-NE4, EHE-NE5, EHE-NE14, EHE-NE20, PBS4, Mugdale 5, TAM-NE47, FC1, Kendall, 94–5224, which had not been sequenced, were included in the PFGE analysis and these strains could be placed within the clade structure established for the sequenced strains. Most of the pathogenic strains examined clustered into three PFGE profiles (Fig. 10), supporting the designation of strains into these pathogenic clades.

## Discussion

In this study, comparative genomic analysis showed that pathogenic strains of *C. perfringens*, which were isolated from birds with necrotic enteritis and contain the *netB* gene, can be classified into two distinct groups. The pathogenic clades are pathogenic clade 1, containing most *netB*-encoding strains and which is separated into subclades 1A (CP4-like) and 1B (NE18-like), and pathogenic clade 2, containing JGS4143/WER-NE36-like strains. Clades 3 and 4 comprise non-pathogenic isolates.

The presence of highly conserved clades of pathogenic necrotic enteritis-causing strains across distinct geographical locations and isolated across several decades suggests that there is persistence or transfer of strains between continents or that the isolates have originated from a common source, possibly from within the breeding stock used for global commercial production of chickens. Alternatively, perhaps these strains are under a common selection pressure and therefore retain or lose common genetic loci. These results expand on the existing knowledge of two pathogenic clades [23], and provide evidence that one of these clades appears to be diverging into subclades (1A and 1B). Pathogenic clade 2 is more closely related to non-pathogenic strains. The use of hybridisation methods such as comparative genome hybridization, as in earlier studies [23], would result in some of these distinct subclades being clustered together. The pathogenic clades 1A and 1B fit within the CHG cluster III and our pathogenic clade 2 and non-pathogenic clade 3 fit within CGH clusters I and II, as defined previously [23]. The clustering of clades observed in this study is supported by other published studies, in particular those involving the use of MLST and PFGE to examine strain diversity [34]. The high level of similarity between strains isolated many years apart suggests a strong selective pressure for their genome to be conserved. This observation is particularly true for pathogenic clade 1A, while other strains such as those in pathogenic clade 2 and the non-pathogenic clades are more diverse. In contrast, the high genomic diversity of the non-pathogenic strains is supported by PFGE studies, which is not surprising since healthy chickens are known to harbour a highly diverse *C. perfringens* population [16, 17, 35]. Our results have demonstrated that pathogenicity of necrotic enteritis does not correlate well to core genome phylogeny, or to the majority of variable genetic loci. Plasmids are known to encode the major factors required for virulence, e.g. toxins, but the variability in both plasmid carriage and chromosomal genetic islands show key regions associated to virulence and pathogenesis mechanisms are varied.

Most of the pathogenic poultry strains examined here are classified into pathogenic subclades 1A and B. These isolates share many of the variable chromosomal regions. The most significant regions separating these subclades from other *C. perfringens* isolates are the adhesion-associated loci and the capsule polysaccharide synthesis loci. They share the VR-10B locus [30], supporting the hypothesis that this adhesion locus is chicken specific and is highly prevalent and conserved in necrotic enteritis pathogenic strains. We previously [30, 31] demonstrated through gene deletion and virulence studies that this adhesion locus is a crucial factor for necrotic enteritis disease. Pathogenic clade 1 has a higher level of chromosomal variation compared to the other clades, hence the separation into subclades 1A and 1B. This variation is mostly due to the integration of various prophage genomes and other smaller genomic variations such as the presence of the variant motility locus and the type I RMS system. All strains within subclade 1B carry a capsule locus encoding proteins that catalyse UDP-mannose additions to the polysaccharide capsule. This region is also shared by approximately half the strains in subclade 1A, while the other 1A strains only encode proteins that facilitate dTDP-rhamnose and choline additions. Plasmid content also differentiates the subclades; clade 1A strains are the only pathogenic strains to carry four plasmids including the *tpeL*-encoding plasmid while clade 1B strains carry three plasmids, which encode Beta2 toxin production, tetracycline resistance and NetB toxin production. Pathogenic clade 1 appears to be diverging and evolving from a common ancestor into the two most prevalent necrotic enteritis-causing populations. From our analysis, we have noted that pathogenic clade 2 strains contain a more variable chromosome compared to the other pathogenic strains. Strains within this clade encode the VR-10A adhesion-associated locus and the simple galactose/glucosamine-based capsule locus. Other regions associated with pathogenic clade 2, and not found in other pathogenic strains, include those that encode genes responsible for L-fucose metabolism and a bacteriocin/antibiotic associated locus.

Pathogenic clade 2 is clustered closely by gene content to many non-pathogenic strains and non-chicken isolates. Therefore, it is hypothesised that pathogenic clade 2 strains were not originally pathogenic chicken strains, but have been introduced into chickens from other sources, and that the strains have subsequently inherited the virulence plasmids through horizontal gene transfer, resulting in the emergence of new strains capable of causing necrotic enteritis in chickens. In *C. perfringens* horizontal gene transfer in the laboratory has been demonstrated to convert strains to different toxin types and disease causing capability [7, 36, 37] so it is possible that this process can also occur in the environment or in the chicken gastrointestinal tract.

It is common to isolate *netB* negative strains of *C. perfringens* from chickens suffering necrotic enteritis [25, 38–41]. It is considered that these strains are either normal or transient flora or isolates in which the plasmid has been lost during initial recovery; seven such *netB* negative isolates are included in this analysis. Comparison of the chromosomes of these strains reveals a degree of similarity to pathogenic clade 2 strains, but with more individual variation. PFGE fingerprinting revealed that each strain has a unique fingerprint, providing evidence that they originate from different backgrounds or have a high level of genomic rearrangements. There is a relatively high degree of genomic variation within all the non-pathogenic chicken strains whether they were isolated from diseased birds or healthy birds, suggesting a high level of divergence or genetic drift, partly driven by the integration and mobilisation of mobile genetic elements that are prolific within the *C. perfringens* species.

Strains isolated from healthy birds with no signs of necrotic enteritis are believed to be commensal or transient *C. perfringens* strains. Both the sequence data and PFGE indicated that most of these strains did not contain large clostridial plasmids. These strains have no association with necrotic enteritis, and lack the *netB* gene. By comparative chromosome analysis, it was seen that several of these strains, including PBD1, PBD5 and PC5, have highly divergent chromosomes compared to other chicken strains. Strains SAF-1 and 11 are an exception; even though these strains lacked large plasmids, they clustered much closer to pathogenic clade 2 and sat within the group of strains that were isolated as *netB* negative from diseased birds. The genetic diversity of these healthy bird isolates suggests that further sampling and sequencing of different *C. perfringens* isolates from healthy individuals may further expand the known diversity of *C. perfringens* genomes. This may help to inform our understanding of strain evolution, conjugative transfer of toxin plasmids, pathogenesis, and normal flora-host relationships.

The ability of strains to adhere to and colonise the host is essential for virulence in many disease systems [23, 31, 42]. The presence of many different capsular biosynthesis and adhesion-associated regions in pathogenic chicken strains suggests that there may be various mechanisms by which strains can cause disease. For necrotic enteritis to occur, expression of the NetB toxin is required, however, a pathogenic strain also needs to compete with other microbes within the gut environment of the chicken host [43]. The presence of the VR-10B adhesion allele is strongly associated with necrotic enteritis [23, 30, 44], suggesting that this allele may provide a selective advantage for colonisation in chickens. The other major alternative allele, VR-10A, is highly mutated in many chicken pathogenic isolates, suggesting that this locus is not required for colonisation or disease in chickens.

In other Gram-positive bacteria capsules play an important role in the disease process [39–45] Three major capsule types were observed in pathogenic strains. Capsules are likely to be a virulence factor as capsule has been implicated in various diseases, with particular roles in colonisation and immune evasion [45–47]. The capsule loci encode proteins with varied UDP-sugar biosynthesis and glycosyltransferase functions, suggesting that very different capsule polysaccharide chains may be synthesised by different strains. Different polysaccharide capsules have been characterised by NMR in *C. perfringens* [48–51]. A recent study characterising the polysaccharide structures in ATCC13124 demonstrated distinct polymer structures containing β-mannosamine, rhamnose, glucosamine and phosphocholine linkage [52], this supports our characterisation of the capsule loci, as ATCC13124 contains the corresponding genes within the CpCAP. Using *Streptococcus sp*. as a model we predict that the conserved genes on both the 5′ and the 3′ end of the locus produce the minimum polysaccharide capsule [45]. Typically most of the enzymes necessary for polysaccharide synthesis are encoded within polysaccharide-specific loci, with many of the nucleotide diphospho-sugars (NDP-sugars) common to other cellular pathways and obtained from cellular pools without the need for the involvement of unique enzymes [46]. Therefore, some difficulty arises in estimating the structure of the polysaccharide capsule without physical examination, although initial studies into structure have been performed by NMR spectroscopy, and mechanical isolation with agglutination tests using immune sera [48–50, 53].

Allelic replacement of capsule regions within and between bacterial species could explain the dramatic amount of capsule and serotype variation observed in bacterial species such as *Staphylococcus sp.* and *Streptococcus sp.* [45, 54, 55] . It is likely that genetic exchange can occur between bacterial species that persist in the same environment as *C. perfringens.* Many other species co-exist in the gastrointestinal tract of animals and so it is potentially an environment of high genomic exchange, which could result in the transfer and exchange of CpCAP regions [45, 46, 56, 57].

It has been proposed [43] that bacteriocin production may be one of the factors responsible for the typical single strain dominance [58] in necrotic enteritis cases. It has been demonstrated that co-challenge with pathogenic and non-pathogenic *C. perfringens* strains resulted in the displacement of non-pathogenic strains from broilers, an important step in the pathogenesis of necrotic enteritis [32, 43, 59]. Therefore, bacteriocin production can be regarded as a potentially important virulence-associated factor. In the isolates in this study the bacteriocin perfrin (*cpp*) is encoded by an integrated phage regions and has similarities to other phage encoded inhibitory proteins [60].

Finally, the presence of a large variety of prophage elements is not only an important factor in establishing phylogenetic relationships between strains, but they can also be an important source of virulence factors [61]. In other A-T rich genomes, such as those of *Staphylococcus aureus* and *Streptococcus pyogenes*, phage integrations account for a substantial amount of the inter-strain genetic variations that are seen [62, 63]. As shown here, and in previous *C perfringens* genome papers, prophages account for a large proportion of the variation seen between *C. perfringens* genomes [14, 23]. In the clostridia, prophages have the potential to manipulate the regulation of genes through their insertion into genes involved in sporulation and metabolism [64]. Most of the prophages found to be associated with *C. perfringens* belong to the family *Siphoviridae* of the order *Caudoviridales* [64–67]*.* Several unique prophages have been found in previous sequenced strains such as ATCC13124, strain 13, ATCC3626, and SM101, as well as in many strains not investigated in this study [14, 64, 68, 69]. Some strains have a single prophage integration, while other strains have up to eight different integration events. These high levels of variation caused by prophage integrations are likely to have a strong influence on *C. perfringens* by potentially introducing new and varied virulence factors such as bacteriocin genes and/or affecting gene regulation. Bacterial immune systems were also characterised including type I and type II restriction modification systems and a CRISPR.

Representative non-chicken strain sequences, accessed from the public databases, were used to compare the variation observed within the necrotic enteritis-associated *C. perfringens* to that seen in the wider population of *C. perfringens* from other sources, including strains of different toxinotypes. Interestingly, a recent WGS study has concluded, based on the carriage of know virulence factors, that the pathogenesis of *C. perfringens* caused necrotic enteritis in turkeys is likely to be different to that seen in chickens [70]. The analysis of non-chicken derived *C. perfringens* isolates suggested that regardless of host, toxinotype, and plasmid content, the non-chicken strains are more closely related to each other and to the non-pathogenic clade 3 chicken isolates and pathogenic strains in clade 2 than to the strains of pathogenic clade 1. Since the toxinotyping system for classifying *C. perfringens* is dependent upon plasmid-determined toxin production [2]*,* the removal of plasmids from typing studies would result in every *C. perfringens* strain being classified as toxinotype A. Therefore, the lack of diversity in these non-pathogenic strains may indicate that extra-chromosomal elements such as plasmids are a major source of genomic variation in non-chicken strains. Another possible explanation for the lack of diversity in non-chicken isolates may be that the strains that can cause disease in mammals share common traits for colonisation and pathogenesis, as mammals are likely more structurally and biologically like one another than to chickens and other poultry in terms of their digestive physiology [71]. Increasing the sampling and sequencing of isolates that are implicated in other diseases, together with the addition of non-pathogenic strains from other animals, will reveal more about the chromosome variation in isolates from other toxinotypes and diseases. Perhaps there is a greater diversity such as that observed in chicken isolates for each different animal host, a suggestion which can only be tested by investigation of much larger numbers of diverse isolates.

## Conclusion

Overall, the current study has shown that necrotic enteritis-causing isolates of *C. perfringens* group into several pathogenic clades and subpopulations based on their chromosomal gene content. This study has demonstrated that pathogenicity does not correlate with core genome content, but rather is correlated with accessory gene content located on the chromosome and plasmids. This study has identified several variable genomic loci with potential roles in virulence that could contribute to alternative pathogenesis mechanisms in different isolates of NetB producing necrotic enteritis causing strains of *C. perfringens*. The variation in the capsule loci, adhesion-associated locus, and prophage elements accounts for most of the chromosomal variation between strains. The presence of virulence and antibiotic resistance plasmids was also shown to be variable between clades. It will be of interest to determine whether any of these loci provide increased fitness or provide a selective advantage of one pathogenic clade over another, as all subtypes appear to persist in the environment, temporally and spatially. In summary, these results suggest that *netB*-positive, necrotic enteritis-causing strains have evolved via several different mechanisms, including clonal expansion and horizontal gene transfer.

## Methods

### Strain collection

The collection of 36 *C. perfringens* strains selected for whole genome sequencing (WGS) included isolates from a range of international locations, isolation dates and host disease status. Their isolation and previous characterisations are detailed in the references listed in Table 1. Diverse strains were selected based on laboratory tests and virulence in a chicken model of necrotic enteritis induction as reported in previous published studies. The common finding is that only isolates that carry the *netB* gene can reproducibly produce disease. We have therefore classified strains that have not been directly tested in a disease induction model as either virulent or avirulent based on their carriage of *netB*. The dataset was supplemented with the addition of 20 WGS from published sequences available in GenBank (Table 1).

### DNA extraction and genome sequencing

Strains were cultured from -80 °C glycerol stocks on fresh Tryptone-Sulphite-Cycloserine (TSC) plates (Oxoid). To prepare genomic DNA for Roche/454 and Illumina MiSeq library preparation single colonies were used to seed overnight cultures in Tryptone-Peptone-Glucose (TPG) broth (5% (*w*/*v*) Bacto-Tryptone (BD Biosciences), 0.5% (w/v) proteose peptone (BD Biosciences), 0.4% (w/v) glucose and 0.1% (w/v/) sodium thioglycolate). The overnight broth was used to seed a fresh TPG broth and incubated for 8 h. Cultures were then centrifuged at 14,000 rpm for 30 min and the supernatant was discarded. The pellets were then subjected to a phenol/chloroform DNA extraction. Sequencing libraries were prepared for Roche/454 sequencing using the NEBNext Quick DNA Library Prep Reagent Set (New England Biolabs) and for Illumina MiSeq using the Nextera XT DNA Library Preparation Kit (Illumina). Genomes were sequenced using Roche/454 (Titanium chemistry) and Illumina MiSeq (2 × 300 bp paired-end reads) methods. Roche/454 read data were assembled using Roche GS De Novo Assembler v2.6 with default settings. Illumina MiSeq read data were quality trimmed using EA-utils v1.1.2 fastq-mcf program [72]: sequence with an average quality score lower than 15 in a 5 bp sliding window was trimmed. Reads with lengths less than 50 bp or more than 70% low complexity were discarded. MiSeq reads were assembled using IDBA-UD v1.1.1 with default settings [73]. For PacBio sequencing, EHE-NE18 was grown overnight as previously described, 10 μl was subcultured into five 1.5 ml Heart Infusion broths (Oxoid) for 3 hours until mid-log phase. Each culture was subjected to the MagAttract HMW Kit (Qiagen) as per the manufacturers’ instructions. The quality of purified DNA was assessed using the Qubit 2.0 (ThermoFisher Scientific) and the High Sensitivity dsDNA kit as per the manufacturer’s instructions. 20 Kb size selection was done using BluePippin Size Selection (Sage Science) and DNA was prepared for sequencing using the SMRTbell Template Preparation Kit 1.0 (Pacific Biosciences) as per the manufactures’ instructions. The genome was sequenced on an RS2 instrument (PacificBioSciences). The RS2 read data were assembled using the Canu Assembler v1.3 with the following options: mhapMerSize = 21′, ‘genomeSize = 3.5 m’, ‘OvlMerSize = 21′, and ‘-pacbio-raw’ [74]. NCBI Blast was used to align the beginning and end of the molecule and matching sequence was detemined from the beginning of the molecule. Seqret (EMBOSS v6.6.0.0) was used to reformat the circulised file to appropriate FASTA format. The genome was then polished using blasr version 5.2.b99b47c (pitchfork release, 10/6/16 PacificBioSciences, 2015). The genome was then subjected to short read error correction using Pilon v1.18 using the MiSeq sequencing data [75]. The whole genome sequence data has been deposited at DDBJ/ENA/GeBank under BioProject PRJNA422745.

### Bioinformatic analysis

The program, HARVEST tools, including Parsnp v1.2, was used to align the core genomes of *C. perfringens* strains and to identify SNPs [76]. This software was designed to identify regions of recombination and thus avoid mis-calling of SNPs; we tested program runs with and without this feature (option ‘-x’) and found no difference in results. Default program settings were used for all other options. To ensure accuracy, the program Snippy v.3.2 (Github.com/tseemann/snippy) was also used to establish core genome SNP phylogeny. Phylogenetic trees of maximum likelihood were created using the RAxML v 8.0 using the general time reversible model with gamma distribution and a 1000 bootstrap replicates [33].

For the pangenome analysis, coding DNA sequences (CDS) were identified in assembled genomes using Prokka v1.11 [77] set to ‘fast’ mode with no rRNA or tRNA detection. Plasmid CDS were removed from the data by first creating a database of *C. perfringens* plasmid sequences from the NCBI nucleotide database using NCBI E-utilities [78]. Command = “esearch -db nucleotide -query ‘(*Clostridium perfringens* plasmid complete sequence) AND “*Clostridium perfringens*”[porgn:__txid1502] NOT (chromosome)’ | efetch -format fasta > cp_ncbi_plasmids.fasta”. Plasmid CDS were then identified using Prokka as described for genomes and made into a BLAST protein database (BLAST v2.2.30). Genome CDS were searched against the plasmid database using blastp. All CDS with an alignment e-value of 1 × 10^− 10^ or less were removed from the genome’s CDS file using a python script (pullplasmids_cds.py, available on GitHub: https://github.com/tallnuttcsiro/pullplasmids_cds.py). The presence of CDS among strains was assigned using cluster analysis. All CDS protein sequences were placed in a single FASTA file and analysed using the UCLUST algorithm of the USEARCH program [29] with a 60% identity radius for clusters (i.e. proteins with 60% alignment identity or greater were clustered as a single CDS around a ‘centroid’ CDS). A full example of linux commands and the associated scripts used is available: https://github.com/tallnuttcsiro/pangenome_using_uclust). Note that the clustering threshold of 60% chosen here is arbitrarily used as a threshold below which we assume non-homologous CDS will not be scored as present among strains. Increasing this threshold gives a lower probability that non-homologous CDS will be amalgamated, whereas raising the threshold divides truly homologous CDS into spurious subdivisions. We found that 60% provided a reasonable compromise, giving a realistic number of pangenome CDS in the sequenced genomes. Our analysis was also confirmed using the Bacterial Pangenome Analysis Pipeline (BPGA) v1.3.0 [79], which also applies the USEARCH algorithms to determine evolutionary relationships. Using the same thresholds on the chromosomal sequences of our dataset provided the same evolutionary relationships. KEGG and COG analyses on the core, accessory and unique genes were performed using the BPGA software [79]. Maximum likelihood tree of the pangenome was created with RAxML v 8.0 using the binary pan matrix as the input and gamma model of rate heterogeneity with 1000 bootstrap replicates [33].

Prophage regions within the assembled chromosome of *C. perfringens* were characterised and located using PHAST (PHAge Search Tool) http://phast.wishartlab.com [80] and further expanded manually. Other variable regions were examined manually and blastn was utilised to examine the presence or absence of these loci within our strain database.

### PFGE fingerprinting

*C. perfringens* strains were grown overnight in 6 ml of TPG broth. Bacterial pellets were collected by centrifugation at 14,000 rpm and the cells were resuspended in 1 ml of 1% PFGE-certified agarose and adjusted to an OD_600_ of 0.8. Agarose plugs were incubated for 1 h with gentle shaking at 37 °C in lysis buffer (0.5 M EDTA pH 8.0, 2.5% of 20% N-lauryl-sarkosyl, 0.25% lysozyme) and subsequently incubated in 2% proteinase buffer (0.5 M EDTA pH 8.0, 2.5% of 20% N-lauryl-sarkosyl 0.25% Proteinase K) overnight at 55 °C. For each isolate, a plug was equilibrated in 200 μL of CutSmart restriction buffer (NEB) at room temperature for 20 min and then digested with 20 U of *Sma*I at 25 °C overnight. Electrophoresis was performed in a 1% PFGE-certified agarose gel and separated with a CHEF-DR-III PFGE system (Bio-Rad Laboratories) in 0.5× Tris-borate-EDTA (TBE) buffer at 14 °C at 300 V for 20 h with a ramped pulse time of 4 to 38 s at an angle of 120°. Gels were stained with GelRed (Biotium) and visualized using UV light. Mid-range and Low-Range PFGE markers (New England Biolabs) were used as molecular DNA ladders. PFGE gels were analysed using GelCompare II software v 6.6 (Applied Maths). Band matching was performed using a 1% position tolerance and 0% optimization factor, and cluster analysis was performed using the Dice similarity coefficient and unweighted pair group method with arithmetic mean (UPGMA).

## Abbreviations

CDS: Coding DNA sequence
CGH: Comparative genome hybridization
CpCap: Capsular polysaccharide synthesis locus
CRISPR: Clustered regularly interspaced short palindromic repeats
MLST: Multi-locus sequence type
NCBI: National Centre for Biotechnology Information
NMR: Nuclear magnetic resonance
ORFs: Open reading frames
PCA: Principal component analysis
PFGE: Pulsed-field gel electrophoresis
RMS: Restriction modification system
SNP: Single nucleotide polymorphism
TPG: Tryptone peptone glucose
TSC: Tryptone sulphite cycloserine
WGS: Whole genome sequencing
